# Tumour angiogenesis normalized by myo‐inositol trispyrophosphate alleviates hypoxia in the microenvironment and promotes antitumor immune response

**DOI:** 10.1111/jcmm.16399

**Published:** 2021-02-23

**Authors:** Bouchra El Hafny‐Rahbi, Klaudia Brodaczewska, Guillaume Collet, Aleksandra Majewska, Krzysztof Klimkiewicz, Anthony Delalande, Catherine Grillon, Claudine Kieda

**Affiliations:** ^1^ Centre for Molecular Biophysics UPR CNRS 4301 Orléans CEDEX 2 France; ^2^ Laboratory of Molecular Oncology and Innovative Therapies WIM Warsaw Poland; ^3^ Postgraduate School of Molecular Medicine (SMM) Warsaw Medical University Warsaw Poland; ^4^ Department of Biophysics Faculty of Biochemistry, Biophysics and Biotechnology Jagiellonian University Kraków Poland

**Keywords:** angiogenesis, cancer, hypoxia, immune response, microenvironment, myo‐inositol trispyrophosphate, oxygen partial pressure (pO_2_), vessel normalization

## Abstract

Pathologic angiogenesis directly responds to tumour hypoxia and controls the molecular/cellular composition of the tumour microenvironment, increasing both immune tolerance and stromal cooperation with tumour growth. Myo‐inositol‐trispyrophosphate (ITPP) provides a means to achieve stable normalization of angiogenesis. ITPP increases intratumour oxygen tension (pO_2_) and stabilizes vessel normalization through activation of endothelial Phosphatase‐and‐Tensin‐homologue (PTEN). Here, we show that the tumour reduction due to the ITPP‐induced modification of the tumour microenvironment by elevating pO_2_ affects the phenotype and properties of the immune infiltrate. Our main observations are as follows: a relative change in the M1 and M2 macrophage‐type proportions, increased proportions of NK and CD8^+^T cells, and a reduction in Tregs and Th2 cells. We also found, in vivo and in vitro, that the impaired access of PD1^+^NK cells to tumour cells is due to their adhesion to PD‐L1^+^/PD‐L2^+^ endothelial cells in hypoxia. ITPP treatment strongly reduced PD‐L1/PD‐L2 expression on CD45+/CD31+ cells, and PD1^+^ cells were more numerous in the tumour mass. CTLA‐4^+^ cell numbers were stable, but level of expression decreased. Similarly, CD47^+^ cells and expression were reduced. Consequently, angiogenesis normalization induced by ITPP is the mean to revert immunosuppression into an antitumor immune response. This brings a key adjuvant effect to improve the efficacy of chemo/radio/immunotherapeutic strategies for cancer treatment.

## 1 INTRODUCTION

Tumour angiogenesis is a direct consequence of hypoxia in endothelial cells present in the tumour microenvironment. The endothelial cell response to hypoxia‐induced signals in a tumour is called the angiogenic switch.^1^ Vessel growth occurs through the recruitment of endothelial cells, by sprouting of pre‐existing vicinal vessels, paracrine action on bone marrow endothelial cell precursors, or other processes, including intussusception, vascular co‐option and mimicry.^2^

All the above‐cited mechanisms are responses to hypoxia, aiming to feed the tumour with nutrients and oxygen through vessel formation in a pathologic microenvironment. The resulting abnormal angiogenesis is a hallmark of cancer, and its functions are deeply impaired.^3^ Inefficient tumour vessels do not ensure sufficient blood flow to increase the oxygen level inside the tumour upon oxygen release by erythrocytes.^4^ Consequently, the tumour remains hypoxic, causing a vicious circle of tumour angiogenic activity and continuous stimulation of proangiogenic pathways. Characterized by the stabilization and transcription of HIFs,^5^ the increased production of factors such as VEGFs, angiopoietin 2 and IL8 maintains a proangiogenic and pathologic microenvironment for tumour cells^3^, ^6^, ^7^ and modulates the autocrine and paracrine effects of the cytokine response towards the tumour.^7^

In this setting, the tumour develops permanent adaptations to the balance of secreted factors and cells. Typically, stromal cells, such as fibroblasts, change their phenotype and become activated, expressing molecules like podoplanin,^8^ which help tumour development.^9^ Cooperation of the tumour microenvironment with tumour cell growth operates through chemokine/chemokine receptor axes, such as CXCL12/CXCR4 and CCL21/CCR7, which respectively recruit immune cell populations which help the tumour growth^10^ and cause the dissemination of metastases in an organ‐specific manner.^11^, ^12^

The tumour microenvironment, therefore, provides optimal conditions for tumour growth, mainly by allowing tumour escape from the immune response.^13^, ^14^, ^15^ Indeed, VEGF‐A is directly produced by tumour cells during hypoxia and is proangiogenic and a potent immunosuppressor. As it circulates, VEGF‐A participates in the recruitment of suppressor cells, such as myeloid‐derived suppressor cells (MDSCs) and Tregs, to the tumour site, where they promote tumour development. Macrophages react to the tumour microenvironment by adopting an M2‐polarized phenotype. M2 tumour‐associated macrophages participate in immunosuppression and cooperate with pathologic angiogenesis.^16^, ^17^ As in hypoxia, tumour cells and immunosuppressor cells, such as MDSCs and tumour endothelial cells, express immune checkpoint molecules like PD‐L and PD‐L2,^18^, ^19^ which can neutralize NK cells and induce CTL tolerization.^20^ Thus, the entry of NK cells and activated CTLs might be impaired, and the activity of these cells may be inhibited.^21^, ^22^

Considering its effects on tumour growth and dissemination, pathologic angiogenesis constitutes an important target for tumour treatment. However, antiangiogenic approaches destroy vessels and create deeper hypoxia and must be avoided as they induce the selection of resistant cancer stem cells.^23^, ^24^

Based on these observations, the ‘non‐antiangiogenesis treatment’ concept arose,^25^ and strategies employing this concept have been designed to normalize tumour vessels rather than eliminate them. Normalization strategies focus on producing new functional vessels, using (a) anticancer drugs, because drugs can more easily reach tumour cells thanks to the re‐established blood flow, (b) radiotherapy, thanks to the elevation of oxygen tension, and (c) immunotherapy, synergistically boosting the immune response.^15^ The main challenge to overcome is to neutralize hypoxia to counteract its most deleterious effects on the tumour microenvironment.^26^ Vessel normalization not only increases treatment efficacy, but also reduces oedema and increases the effects of cytotoxic anticancer drugs. Thus, there are many benefits to considering normalization as an adjuvant strategy.^15^ As opposed to adapted‐angiogenesis‐based therapies, which must take advantage of transiently appearing therapeutic windows,^27^ the possibility of stably normalizing vessels using myo‐inositol trispyrophosphate (ITPP) has to date been the most effective option.^28^ As an allosteric effector of haemoglobin,^29^ ITPP also stabilizes normalized vessels by interacting with and activating endothelial PTEN.^28^, ^30^ This tumour suppressor molecule is a primary regulator of vessel formation and modulates endothelial cell reactivity through the control of immune checkpoints, such as PD‐L1,^31^ through cross controlling NOTCH4.^32^

Myo‐inositol‐trispyrophosphate is a non‐cytotoxic molecule,^28^, ^33^ and its tumour‐reducing effects^30^, ^34^ are suggested to operate through vessel normalization, leading to sustained increases in oxygen partial pressure (pO_2_) which counteracts hypoxia inside the tumour and inverts its effects. This work shows that ITPP‐induced stable normalization induces deep changes in the tumour microenvironment due to hypoxia compensation using two tumour models: melanoma and mammary carcinoma. ITPP‐induced stable normalization further stops the HIF‐dependent cytokinic and cellular responses. As directly demonstrated here, NK cell infiltration and its antitumour activity are impaired by PD1 binding to PD‐L1 expressed on hypoxic endothelial cells in the tumour, and this effect is reduced by ITPP treatment. This treatment mainly inverts immunosuppression—the key reason why current therapies fail. Here, we report for the first time a strategy that fulfils the main conditions required to increase the efficacy of cancer treatment. Indeed, this approach addresses the present challenges facing cancer treatment, influencing the tumour microenvironment by permitting the recruitment of active immune cells, lowering immune checkpoint activity, and reducing PD‐L1 expression at the endothelial cell level to allow the entry of immune cells into the tumour.

## 2 MATERIALS AND METHODS

### 2.1 Ethics statement

All animal‐related experiments were conducted in accordance with approved guidelines and regulations. Experimental protocols were approved by the French Ethics Committee for Animal Experimentation, CNRS Orleans campus CNREEA 03 Ethics Committee, authorization number CLE CCO 2010‐004.

### 2.2 Mice and tumour models

C57BL/6 mice (C57 Black6 strain from CC Little 1921), BALB/c mice (from Bagg and Albino, 1913), and nude—Rj:NMRI‐nu—mice were obtained from Janvier Laboratory. Animal care and experimental procedures were performed following government and institutional guidelines and regulations and were approved by the Ethics Committee.

The mouse melanoma model in C57BL/6 and Rj:NMRI‐nu nude mice: Murine B16F10 cells were implanted subcutaneously in the leg of C57BL/6 or on Rj:NMRI‐nu mice, as described previously,^30^ by injecting a plug of 10^5^ cells in 100 µl Matrigel (BD Biosciences).

Mouse carcinoma model: 4T1 murine mammary carcinoma (10^4^ cells in Matrigel) cells were injected into the mammary fat pad of BALB/c mice.

### 2.3 Cell lines and cell culture

Murine brain (MBrMEC) and bone marrow (MBMMEC)‐derived organospecific microvascular endothelial cells (ECs) were established as described^34^, ^36^, ^37^, ^38^ and patented (N1170 3915.6, N13/521715). EL4 (ATCC^®^ TIB‐39™) and EL4‐IL2 (ATCC^®^ TIB‐181™) were purchased from ATCC. ECs were cultured in OptiMEM medium supplemented with 2% FBS on Primaria tissue culture‐treated plastic vials (Corning™, VWR). ECs were cultured in OptiMEM supplemented with 2% FBS, and lymphoblasts were maintained in DMEM supplemented with 10% FBS. Upon 80% confluency, ECs were detached with Accutase and used for experiments. All cell culture reagents were purchased from Invitrogen unless otherwise specified.

### 2.4 Hypoxia treatment

Endothelial cells were seeded in 24‐well Primaria plates in duplicate, with 10 × 103 of MBrMEC or 12.5 × 103 of MBMMEC cells per well in 500 μL of OptiMEM medium, and allowed to adhere to the culture surface for 24 hours in a humidified incubator at 37°C with 5% CO_2_. Media were exchanged with normoxia‐ or hypoxia‐equilibrated‐OptiMEM (1% pO_2_ for 48 hours), and cells were placed in either a standard normoxic atmosphere (17.8% pO_2_) incubator or a humidified incubator and hypoxia workstation (X3, Biospherix) at 1% pO_2_ for 48 hours.

### 2.5 Adhesion assay

EL4 and EL4‐IL2 cells were stained with DiO (Molecular Probes) according to the manufacturer's instructions, 24 hours before the addition of ECs. Cells were washed twice with warm hypoxic or normoxic OptiMEM medium, and 0.25 × 10^6^ (ratio 1:5) DiO‐stained EL4 or EL4‐IL2 in serum‐free DMEM were added and incubated for 20 min at 20°C or, when specified at 4°C, with agitation. Non‐adhering cells were washed away with OptiMEM, and the remaining cells were detached by Accutase, washed twice with warm PBS, and stained with V450 viability stain (Becton and Dickinson). Cells were washed, suspended in Stain Buffer with FBS (Becton and Dickinson) and acquired on a Canto II cytometer (Becton and Dickinson). At least 30,000 events were recorded, and only live cells (V450low) were analysed. Leukocyte adhesion was calculated as the ratio of collected EL4 cells (DiOhi) to the number of ECs (DiO negative and low). For inhibition of cell adhesion, a neutralizing anti‐PD‐L1 monoclonal antibody, anti‐PD‐L1Fc silent, chimeric and blocking mouse IgG1, clone alphaPD‐L1 (00377‐1.4BT), was obtained from absolute Antibody, USA, and added to MBMMEC endothelial cells incubated in the culture at 10 µg/mL for 1 hour under hypoxia or normoxia before the adhesion experiments.

### 2.6 ITPP treatment

As described previously,^30^ ITPP was injected intraperitoneally (1.5 g/kg: in saline) twice a week over 3 weeks. Injections began on day 7 and were repeated on day 8 post tumour inoculation (day 0). Serial treatments were applied on days 15 and 16, and 21 and 22. Tumours were extracted and weighed at the indicated times.

### 2.7 Preparation of single‐cell suspensions

Tumour samples were immediately transferred into PBS on ice. Biopsies were cut into small pieces, then filtered through a cell strainer after collagenase/dispase (Gibco) dissociation. Erythrocytes were eliminated by lysis buffer (eBiosciences).

When specified, tumours were depleted of CD45+ and/or CD31+ cells by magnetic separation (Easy Sep magnet, StemCell Technologies Inc).

### 2.8 Cell staining for flow cytometry

Single cell suspensions were stained with monoclonal antibodies for 1 hour at 4°C. One to four‐color flow cytometry was performed (FACS LSR, Becton Dickinson). Data were acquired using the CellQuest software (Becton Dickinson) using at least 100 000 events gated for live cells.

The following conjugated rat anti‐mouse mAbs were used: CD45‐PerCP, CD11b‐APC, CCR4‐PE, CCR5‐PE, CCR7‐PE, CCR10‐PE and CXCR4‐PE (BD Biosciences); CD49b‐APC (Biolegend); CD226‐PE, CD4‐FITC, CD25‐APC, CD8a‐FITC, PD1‐PE, CTLA4‐PE, PDL1‐PE‐Cy7, PDL2‐FITC, CD47‐APC (eBiosciences), CD11c‐FITC, GR1‐FITC (Miltenyi Biotech), CD206 (Santa Cruz), and anti‐mouse Foxp3 staining set PE (eBioscience).

### 2.9 Cultured cell‐surface marker analysis by flow cytometry

PD‐L1 and PD‐L2 levels were measured in ECs cultured as described above. After a 48 hours exposure to hypoxia, cells were washed with PBS, stained with V450 viability stain (Becton Dickinson) and then stained with APC‐anti‐PD‐L1 or PE‐anti‐PD‐L2 antibodies (Clone: MIH18 and MIH5 (RUO), respectively, from Becton‐Dickinson). Cells were analysed on a Canto II cytometer (Becton‐Dickinson). A total of 30 000 events were acquired, gated on live cells (V450low). PD‐L1 and PD‐L2 levels are expressed as a percentage of positive cells and as mean fluorescence intensity (MFI). NK cells, EL4 cells and EL4‐IL2 cells were labelled by anti‐PD‐1 antibody (clone RMP1‐30, Becton‐Dickinson). For cell adhesion and PD‐L1 inhibition, we used the anti‐PD‐L1Fc silent, chimeric and blocking mouse IgG1, clone alphaPD‐L1 (00377‐1.4BT), from Absolute Antibody, USA.

### 2.10 Immunohistochemistry

Tumour tissues were embedded in tissue freezing medium (Tissue‐Tek; Sakura) and snap‐frozen in liquid nitrogen. Tumour cryosections were fixed and stained with mouse anti‐CD31 (rat monoclonal IgG2a; eBiosciences), anti‐CD49b (Rat IgM; BD Pharmingen), or anti‐Firefly Luciferase (Rabbit IgG; Abcam) or anti SOX2 antibody (Rabbit IgG clone LS‐C108535 LSBio) before tetramethylrhodamine isothiocyanate (TRITC) or fluorescein isothiocyanate secondary (FITC) antibodies were added. Nuclei were stained with bisbenzimide H 33 258 (Sigma‐Aldrich).

### 2.11 ELISA assay for VEGF production

VEGF was measured in RenCa cell culture supernatants (after a 72 hours exposure to hypoxia/normoxia) using a commercially available enzyme‐linked immunosorbent assay that recognizes natural and recombinant mouse VEGF (ELISA, R&D Systems, Minneapolis, USA). The results were normalized to 10^6^ cells.

### 2.12 Quantitative real‐time PCR

Cellular mRNA was extracted using the RNeasy Plus mini kit (Qiagen). mRNA was eluted in RNase‐free water. Absorption spectra were measured on an ND‐1000 spectrophotometer (NanoDrop Technologies, Wilmington, DE) before storage at −80°C. RNA was reverse‐transcribed to cDNA using the ‘Maxima First Strand cDNA Synthesis kit for RT‐qPCR’ (Fermentas); 3 µg of RNA was used for each sample. The obtained cDNA was stored at −20°C before qPCR. Real‐time PCR was performed on a LightCycler 480 (Roche) using SYBR Premix Ex Taq (Perfect Real Time) (Takara) and QuantiTect Primer Assay (Qiagen) in white 96‐well optical microtiter plates (Roche). All reactions were performed in triplicate and reported as average values. For reference, seven housekeeping genes were tested. Means and standard deviations were calculated, and the gene which had the lowest standard deviation was chosen as the reference. For each target gene, the mean and standard deviation were calculated and then normalized by the corresponding value for the reference gene (*ppia*), to obtain the ΔCp.

### 2.13 Real‐time PCR for vegf‐a and vhl

Total RNA was extracted from cells using TRIzol and the RNA extraction kit Direct‐zol RNA Miniprep Plus (Zymo Research). RNA quality and concentration were determined by measuring the absorbance at 230, 260 and 280 nm using μDrop in a Multiskan™ GO microplate spectrophotometer (Thermo Fisher Scientific). SuperScript IV VILO Master Mix (Thermo Fisher Scientific) was used for cDNA synthesis, with a DNase step. The relative mRNA expression levels of *vegf* and *vhl* were obtained by real‐time quantification PCR using the TaqMan PCR Master Mix (Applied Biosystems) and TaqMan assay primer sets (*vhl*‐Mm00494137, *vegfa*‐Mm00437306, Applied Biosystems, Foster City, California, USA), and the CFX96 TouchTM Real‐Time PCR Detection System (BioRad). Data were analysed using the 2(−delta delta c(t)) method, with normalization to the expression of *act‐b* (Mm02619580) as a house‐keeping gene.

### 2.14 Oxymetry measurement of the tumour and effect of treatment with ITPP

Oxymetry imaging was done by the photoacoustic method at day 7 after 4T1 tumour implantation (5 × 10^5^ cells in the mammary fat pad), on a locally shaved area using depilatory cream and anaesthetic. Isoflurane (Isovet, Virbac) was used at 3.5% for 3‐60 seconds, then 1.5% along the imaging process adjusted to a breathing frequency of 50/min. Mice were then placed on the heating pad of the VEVOLAZR Imaging Station (FUJIFILM VisualSonics) for in vivo imaging. Images were recorded in the OxyHemo mode, which collected data at 750 nm (Hb‐tot) and 850 nm (Hb‐O_2_) to create and display a parametric map of estimated oxygen saturation using the LZ400 probe at 30 MHz. The saturation of oxygen (SO_2_) in the tumour was followed before and after injection of ITPP. The saturation values corresponded to an average of sO2 values on the 3D tumour volume using a step size of 0.102 mm.

Blood flow was assessed by Laser Doppler (Oxy Flow).

### 2.15 Statistical analysis

All data are expressed as means ± SEM. In vitro cell data are expressed as means + SEM. All statistical analyses were performed using GraphPad Prism 7.0 software. In adhesion and flow cytometry in vitro, the experiment data represent the means of three biological replicates in triplicate, *P < .05 using the Mann‐Whitney *U* test.

Flow cytometry experiments data are represented by the means ± SEM of N representative experiments. Statistical significance was calculated by Student's *t* test (Microsoft Excel). *P* values were determined by Student's *t* test. A *P* value of less than 0.05 was considered statistically significant. *Indicates statistically significant differences (*P* < .05), ** indicates *P* < .01 and *** indicates *P* < .005.

## 3 RESULTS

### 3.1 Tumour vessel normalization through ITPP treatment and tumour pO_2_ status

**FIGURE 1:**
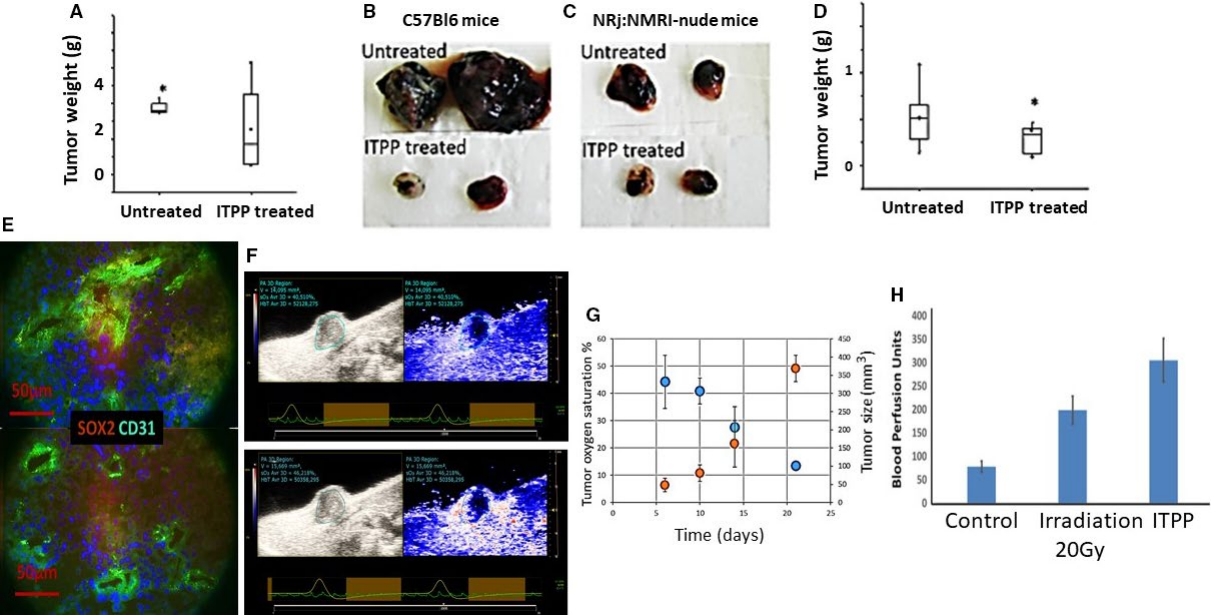
Evolution of melanoma and mammary carcinoma tumour growth and microenvironment composition upon treatment by ITPP. A, C57 BL6 mice received 10^4^ B16F10‐Luc cells, were treated by ITPP to reach vessel normalization until day 21 and were weighed on day 23. Data represent the means of seven experiments (*P < .05); B, Examples of the biggest and smallest tumors obtained for B16F10 Luc cells in C57BL/6 mice, in the absence of treatment (upper panel) and upon serial treatment by ITPP (lower); C, Examples of the biggest and smallest tumours obtained for B16F10 Luc cells in NRj:NMRI‐nude mice, in the absence of treatment (upper panel) and upon serial treatment by ITPP (lower); D, BALB/c‐by mice received 10^5^ 4T1 cells and were treated by ITPP to reach vessel normalization until day 21, and the tumours were weighed at day 31. Data represent the median values of five experimental mice from three separate experiments (*P < .05); E, Immunofluorescent labelling of B16F10 tumours at day 23 for SOX2 marker and CD31 before (upper panel) and after ITPP treatment (lower panel). F, A typical example of photoacoustic measurements and dynamic change in oxygen saturation (sO2 average in %) after 7 days of growth of 4T1 murine mammary carcinoma cells, upon treatment by ITPP injected intraperitoneally. The left panel corresponds to 4T1 tumour oxygen saturation and tumour size in control conditions at day 7. The right panel represents the 3D photoacoustic imaging the same 4T1 tumour 60 mins after intraperitoneal ITPP injection. G, Evolution of the 4T1 tumour oxygenation and growth, along the treatment by ITPP according to the described protocol. H, Effect of ITPP treatment on blood flow perfusion assessed by Laser Doppler in the tumour in comparison with the control blood perfusion effect obtained by 20 Gy radiation. Data are expressed as the mean + SEM of Blood Perfusion Units‐BFU on groups of seven mice from three separate experiments

When tumour‐bearing animals were treated with ITPP under conditions that have been previously shown to normalize vessels,^30^ a reduction in tumour size was observed (Figure 1).

Typical examples are shown for both normal and nude mice for B16F10 melanoma (Figure 1A‐C) and 4T1 mammary carcinoma (Figure 1D) cells. ITPP was shown to be non‐toxic for both animal types^30^ and cells treated separately.^30^ Applying the protocol that we have described previously,^30^ our hypothesis regarding the influence of pO_2_ changes in the tumour microenvironment was assessed. Figure 1E shows the reduction of stemness associated SOX2+ cells^35^ upon ITPP treatment, together with a deep change in the vessel‐associated structures before treatment which appear in a distinct localisation and as regular vessel structures after ITPP treatment. Indeed, from chaotic CD31‐expressing endothelial cells (upper panel) in non‐treated tumours, upon ITPP treatment, the vessels displayed CD31+ endothelial cell organization (lower panel). The effect of ITPP treatment on oxygen partial pressure inside the tumour was confirmed by real‐time measurement of the oxygen saturation inside the tumour, by photoacoustic assessment over up to 70 minutes after intraperitoneal injection of ITPP, as described^36^ (Figure 1F), and the evolution of the oxygen saturation inside the tumour was followed along the treatment (Figure 1G). Moreover, the effect of ITPP on the vessel's functionalization was controlled in the applied protocol in comparison with the known effect of irradiation on increasing the blood flow, as measured by Laser Doppler (Figure 1H), showing that ITPP treatment following the previously defined protocol was more efficient than 20 Gy irradiation at increasing blood perfusion.^37^ The immune response^38^, ^39^ was tested, and we found a deep influence of pO_2_ changes on the cytokine composition of the tumour microenvironment.^30^

The immune reaction was studied when ITPP‐treated tumours were half the size of the controls.

This point was reached 23 days after injection with 10^4^ B16F10 cells, both in C57BL/6 and Rj:NMRI‐nu nude mice. It was reached after 31 days with 10^5^ injected 4T1 cells in BALb/c‐by mice. Treatments were stopped on day 21 for 4T1 cells, and 50% tumour growth was observed at day 23 for melanoma cells. An equivalent reduction was not observed in mammary carcinoma cells until after the arrest of treatment at day 31 (Figure 1F).

### 3.2 Effects of ITPP treatment on the tumour microenvironment‐NK cell response

**FIGURE 2:**
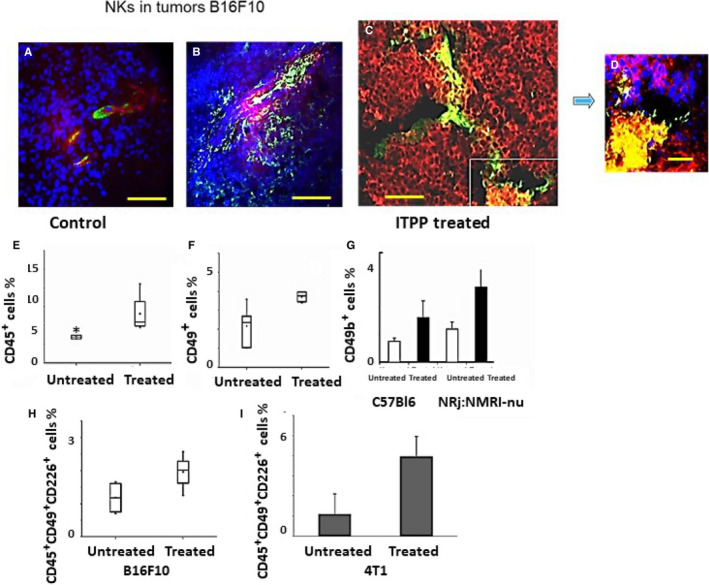
Influence of ITPP treatment of tumours on NK cell recruitment and activation. B16F10 Luc tumours extracted on day 23 were labelled for CD49b+ and CD31+ cell detection (A, B) and for Luc+ expressing cells (C, D). Nuclei were detected by DAPI (A, B, D). Bar = 100 µm (A, B); bar = 40 µm (C); bar = 10 µm (D), pictures are taken with a Zeiss Axiovert fluorescence microscope and analysed by the Zeiss Axiovision software. E, Flow cytometry quantification of the immune CD45+ cells before and after treatment with ITPP. F, CD45+ CD49+ NK cell quantification in B16F10Luc tumours upon ITPP treatment. G, Effect of ITPP treatment on NK cell recruitment in B16F10Luc tumours growing in NRj:NMRI nude mice, compared with normal immunocompetent C57Bl6 mice. H, Activated CD45+ CD49+ CD226+ NK cell quantification in B16F10Luc tumours before and after ITPP treatment. I, In 4T1 mammary carcinoma, activated CD45+ CD49+ CD226+ NK cells were detected by flow cytometry before and after ITPP treatment. Data represent the median values (E, F, H) of five experimental mice from three separate experiments and the means of five experimental mice from three separate experiments (G, I) (*P < .05)

The immune cell infiltrate was analysed by immunocytochemical labelling of tumours extracted from the animals on day 23 after tumour cell implantation. NK cells were detected by anti‐CD49b labelling, and endothelial cells by anti‐CD31 labelling (Figure 2A,B). NK cells remained in the vessels of non‐treated tumours (Figure 2A), whereas they infiltrated the tumour mass of ITPP‐treated tumours (Figure 2B). This is confirmed in Figure 2C, which displays the distribution of CD49b+ NK cells among B16F10 cells transfected with the Luciferase gene, and stained with an anti‐luciferase antibody. The insets in Figure 2C,D indicate the co‐localization of NK cells with tumour foci.

The effect of ITPP on NK cell recruitment was quantified by immunocytochemical labelling of the tumour cells and tumour stromal cells from the whole tumour microenvironment and assessed by flow cytometry. Figure 2E shows that the proportion of immune cells (CD45+) in the tumour is higher in ITPP‐treated than in control mice; the number of NK cells was doubled in the tumour site (Figure 2F). The commitment of NK cells to the hypoxia regulation‐induced response was confirmed by the reaction observed in nude mice upon B16F10‐Luc implantation, and treatment with ITPP. In these immune‐deficient mice, the T cell immune response is compromised, whereas the NK cell response is increased.^40^ Figure 2G confirms this effect, showing a higher proportion of CD49b positive cells (1.5 fold) found in the tumours raised in nude mice, compared with normal C57BL/6 mice. This recruitment is similarly enhanced in both strains of mice, as indicated by the proportions of NK cells found in the tumour before and after ITPP treatment (*R* = 2.25).

Moreover, this increase in intratumour NK cells upon ITPP treatment corresponds to an increase in the numbers of activated (CD45+CD49b+CD226+) NK cells, as estimated by flow cytometry after ITPP treatment (Figure 2H) in melanoma‐bearing mice. Those data were completed by fluorescence microscopy assessment showing the increased numbers and the distribution of CD25^+^NK (Figure S1) and CD69^+^NK cells (Figure S2) in the tumour site after ITPP treatment with reference to the CD31^+^ vessels and confirmed by immunohistochemical detection of the CD49b+ NK cells upon such treatment (Figure S3).

This result was confirmed in the mammary carcinoma model with 4T1 cell bearing mice, in which ITPP treatment induced higher recruitment of activated NK cells (Figure 2I).

### 3.3 Intra‐tumour evolution of myeloid‐derived suppressor cells and the phenotype of infiltrating macrophages upon ITPP treatment

**FIGURE 3:**
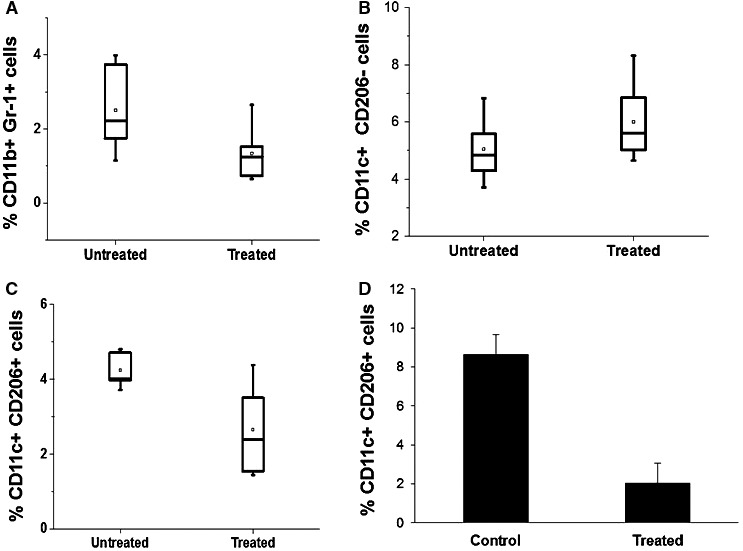
ITPP‐induced reduction of immunosuppressive myeloid‐derived and macrophage cell populations inside the tumour CD45+ cell population (A, B). ITPP treatment reduced the proportions of CD45+ CD11b+ Gr1+ MDSCs in B16F10 Luc melanoma (A) and tended to increase CD45+ CD11c+ CD206− M1 macrophages (B). Data represent the median values of five experimental mice from three separate experiments (*P < .05). (C, D) ITPP treatment decreased CD45+ CD11c+ CD206+M2 macrophages in B16F10Luc melanoma (C). Data represent the median values of five experimental mice from three separate experiments (*P < .05, **P < .01) in 4T1mammary carcinoma (D). Data represent the means of six experimental mice from three separate experiments (*P < .05)

The tumour microenvironment is characterized by the presence of myeloid‐derived immune suppressor cells (MDSCs), which arrive through bone marrow mobilization. These cells exacerbate tumour development and immune escape. In tumours, MDSCs express CD11b and Gr‐1.^41^ ITPP treatment reduced the proportion of MDSCs in the tumour (Figure 3A).

This was accompanied by an increase in the numbers of macrophages expressing the M1 type antigens (CD45+ CD11c+ CD206−) (Figure 3B) indicating that the relative changes in the proportion of cells whose phenotype is compatible with the M1 type, was increased and confirmed by the fluorescence microscopy study showing the increased numbers and intratumour distribution of M1 type of CD68+ cells (Figure S4).

The proportion of macrophages displaying an M2 (CD45+CD11c+CD206+)‐polarized phenotype, which aids tumour immune suppression, was reduced upon treatment with ITPP. This was observed in melanoma (Figure 3C), as well as in mammary carcinoma (Figure 3D).

### 3.4 Evolution of the T cell populations infiltrating the tumour upon ITPP treatment

**FIGURE 4:**
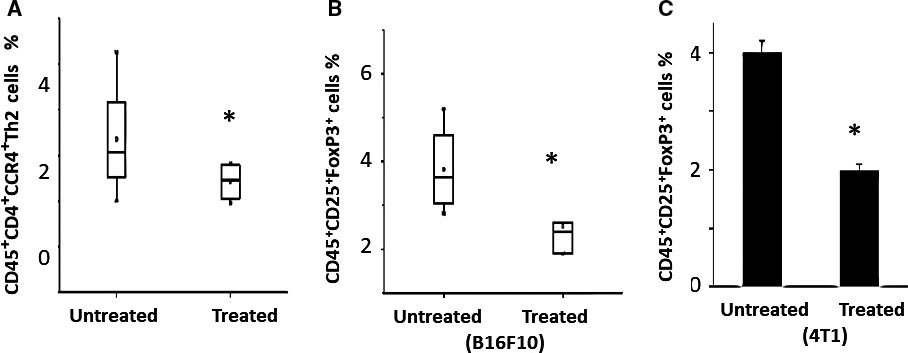
ITPP‐induced reduction of inflammation mediators Th2 cell and immunosuppression mediators Treg cell populations inside the tumour CD45+ cell population (A) ITPP treatment reduced the proportions of CD45+ CD4+ CCR4+ Th2 cells in B16F10 Luc melanoma. Data are median values of five experimental mice from three separate experiments (*P < .05). B, C, ITPP treatment reduced the proportions of CD45+ CD25+ FoxP3+ cells in B16F10 Luc melanoma (B); Data are median values of five experimental mice from three separate experiments (*P < .05), and in 4T1 mammary carcinoma (C). Data represent the means of five experimental mice from three separate experiments (*P < .05)

The proportion of Th2 cells reflects the inflammatory state and is known to affect tumour progression. Th2 cells, characterized as CD45+CD4+CCR4+, were decreased at the tumour site upon ITPP treatment (Figure 4A). Regulatory T cells, which cooperate in tumour development and growth, also clearly responded to ITPP treatment, as the proportion of CD45+CD4+CD25+FoxP3+ Treg cells was significantly decreased in B16F10 Luc tumours when treated with ITPP (Figure 4B). The effect of ITPP treatment on Tregs was confirmed in 4T1 mammary carcinoma‐bearing BALB/c mice (Figure 4C).

### 3.5 The expression of immune checkpoint molecules in the tumour microenvironment is modulated by ITPP treatment

The observed effect of ITPP on reducing the proportion of immunosuppressive cell populations, such as those of MDSCs, macrophages and Tregs, prompted the analysis of the expression of key immune checkpoint molecules: PD1 and its ligands PD‐L1 and PD‐L2.

**FIGURE 5:**
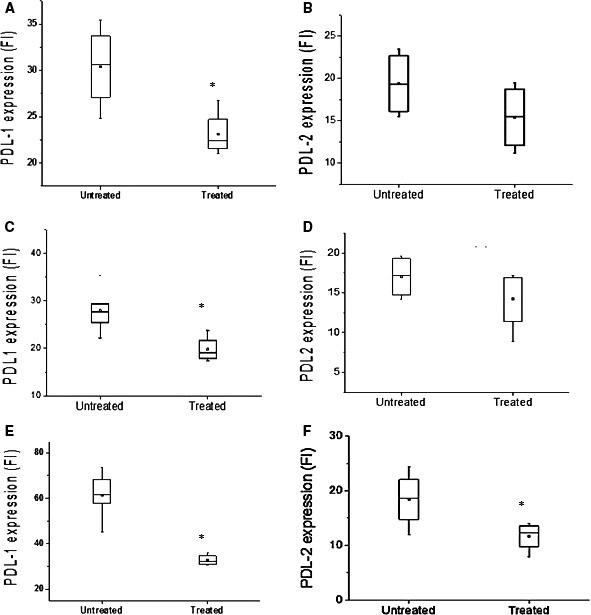
Modulation of the immune checkpoint molecules PD‐L1 and PD‐L2 in the tumour and immune cells upon ITPP induction of tumour vessel normalization. A, B, Dilacerated tumour cells were labelled by anti‐PD‐L1 and anti‐PD‐L2, and their expression analysed by flow cytometry for the total population and gated CD45− non‐immune cells (C, D) and immune CD45+ cells (E, F) populations. Data represent the means of five experimental mice from three separate experiments (*P < .05)

Figure 5 displays the flow cytometric analysis of cells in the tumour (Figure 5A,B) and the distinct immune and non‐immune cell populations, CD45+ (Figure 5C,D) and CD45− (Figure 5E,F), respectively. ITPP treatment induced a clear reduction in the expression of PD‐L1, and to a lesser extent PD‐L2, on the total tumour cell population (Figure 5A,B), as well as on the isolated CD45− cells (Figure 5C,D), and most clearly on the CD45+ immune cell population (Figure 5E,F). As shown, PD‐1 ligand expression was strongly reduced on CD45+ cells but also CD45− cells upon ITPP treatment.

**FIGURE 6:**
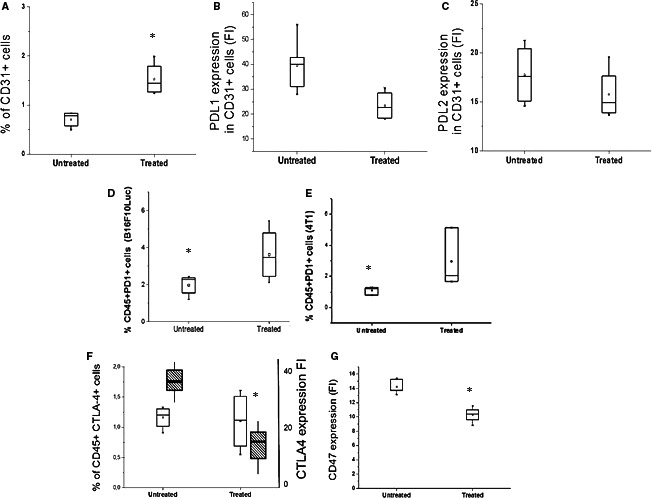
Influence of ITPP treatment on immune checkpoints on distinct cell types in the tumour (A–C) identification of PD‐L1 and PD‐L2 on endothelial cells from a tumour, and modulation by ITPP treatment. Endothelial cells were identified based on their expression of CD31 by flow cytometry (A). The endothelial cell number increased upon ITPP treatment (A). CD31+ endothelial cells expressed less PD‐L1 (B) and tended to express less PD‐L2 (C) after ITPP treatment. Data represent the means of five experiments in triplicate (*P < .05). D, E, The CD45+ immune cell population in the tumour identified by flow cytometry was enriched in PD1‐expressing cells upon ITPP treatment in B16F10 melanoma (D) and 4T1 mammary carcinoma (E). Data represent the median values from five experimental mice and three separate experiments (*P < .05). F, Detection of the CTLA4‐expressing cells among immune cells (CD45+) and level of expression following treatment with ITPP, by flow cytometry. Data represent the means of five experiments in triplicate (*P < .05). G, Detection of the CD47 level expression in B16F10 tumours by flow cytometry. Data represent the median values from five experimental mice and three separate experiments (*P < .05)

Figure 6 shows that when ITPP is used to treat B16F10Luc melanoma‐bearing mice, the proportion of CD31+ endothelial cells is higher than in non‐treated tumours (Figure 6A). CD31 is more highly expressed on endothelial cells in normoxia than in hypoxia and is a junction molecule that strengthens vessels and reduces their permeability.^38^, ^42^ CD31 expression is indicative of vessel normalization.^43^, ^44^, ^45^ Among CD31+ endothelial cells, PD‐L1 was observed to be strongly expressed before ITPP treatment and was considerably reduced in treated tumour CD31+ endothelial cells (Figure 6B). The second PD1 ligand, PD‐L2, was also expressed, although to a lesser extent than PD‐L1, and was reduced by hypoxia alleviation/vessel normalization after treatment with ITPP (Figure 6C). Upon reduction in the PD1 ligands, ITPP treatment may also be accompanied by an increase of immunocompetent cells expressing PD1. As above, the proportion of CD45+ cells was increased by ITPP treatment (Figure 2E). Reduction in the PD1 ligands, especially on endothelial cells, suggests a possible control of the mechanism by which NK cells enter the tumour. Figure 6D,E shows an increased number of CD45+ cells inside tumours that were treated by ITPP. This corroborates the data reported in Figure 2F–I, pointing to an increased proportion of NK cells inside the tumour upon ITPP treatment and increased activated NK cells (CD49b+CD226+),^46^ as well as invasion of the tumour, as opposed to non‐treated tumours where NK cells were found in the vessels (Figure 2A–D). Hypoxia induction of PD‐L1 on endothelial cells was reduced upon ITPP treatment (Figure 6B,C). The Treg CD45+PD1+ cells^47^ proportion was considerably reduced among the whole tumour population, both in melanoma (Figure 6D) and mammary carcinoma (Figure 6E).

The effect of ITPP on other immune checkpoints was also assessed, including CTLA4 expression among CD45+ cells. No significant change was observed in terms of cell numbers, but the level of CTLA4 expression was lowered with ITPP treatment (Figure 6F). The intensity of HIF‐1‐regulated CD47 expression^27^, ^48^ was found to be reduced in tumour B16F10 melanoma cells (Figure 6F). This stem cell marker protects tumours against cytotoxic immune cells, promotes evasion from phagocytosis and maintains cancer stem cells.

### 3.6 Mechanism of NK cell to endothelial cell recognition

The expression of PD‐L1 on the surface of endothelial cells, its induction by hypoxia and the observed extravasation of NK cells made possible by alleviating hypoxia with ITPP raised the hypothesis of direct PD‐1 to PD‐L1 cross‐talk between activated NK cells and PD‐L1+ endothelial cells in the hypoxic microenvironment.

**FIGURE 7:**
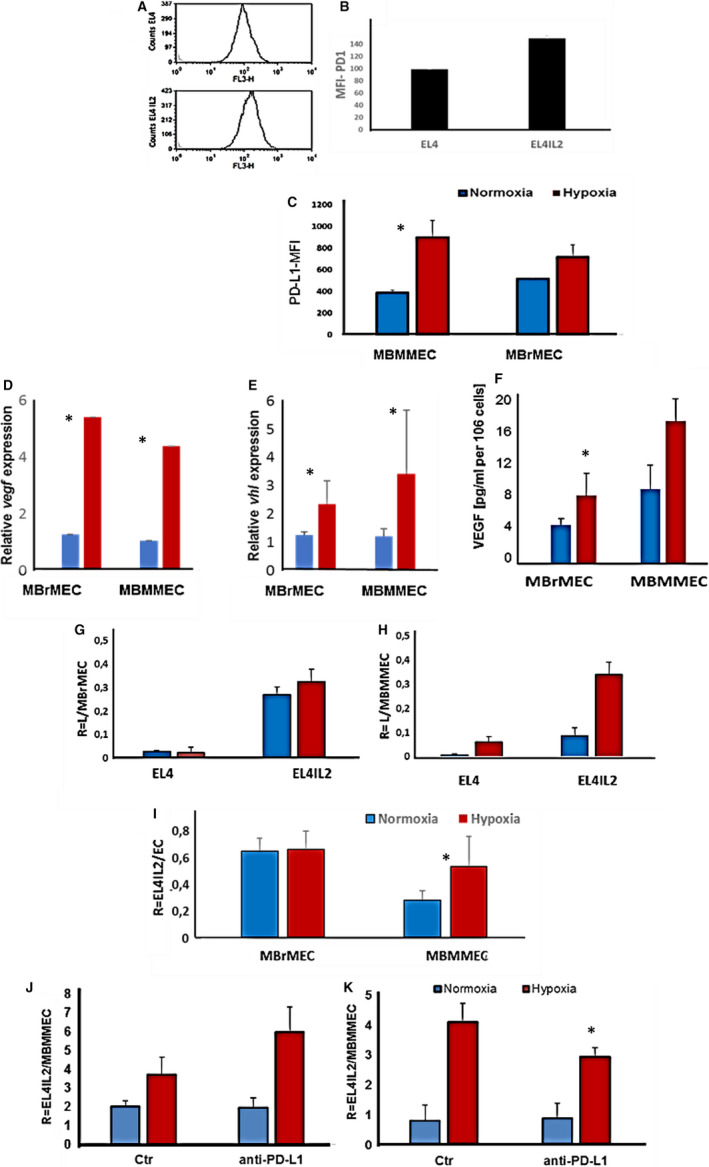
Hypoxia‐mediated adhesion of NK‐to‐microvascular endothelial cell recognition involves immune checkpoint molecules. A,B, PD‐1 detection on NK cells by flow cytometry. EL4 and EL4 IL2 cells labelled with anti‐murine‐PD1 antibodies strongly express PD1. B, Higher expression is detected on activated EL41L2. Means of three biological replicates are shown, *P < .05. C, Effect of hypoxia on surface PD‐L1 expression by ECs, MBMMEC and MBrMEC. Cells were cultured in normoxia (17.8% pO_2_) or hypoxia (1% pO_2_) for 48 h before anti‐PD‐L1 labelling. Staining is expressed as a relative MFI of PD‐L1. Means of three biological replicates are shown, * *P* < .05. D‐F, Effect of hypoxia on the expression vegf and vhl transcripts measured by RT‐PCR (D, E) and VEGF protein production by ELISA (F). Means of three biological replicates are shown, * *P* < .05. G, H, Adhesion of activated EL4 IL2 cells as compared to EL4 cells to organospecific ECs from the brain (MBrMEC) and bone marrow (MBMMEC) under the influence of hypoxia. DiO‐labelled EL4 and EL4 IL2 cells were allowed to adhere in a 5 to ratio to ECs for 20 min in normoxia (pO_2_ = 17.8%) or hypoxia (pO_2_ = 1%). R = number of adhering cells per endothelial cell. *P < .05. I, The selective effect of hypoxia on organospecific ECs’ recognition of the EL4IL2 cells MBrMEC and MBMMEC, cell cultured in normoxia or hypoxia for 48 h. Normoxic, DiO‐stained EL4‐IL2 cells adhered for 20 min. R is the number of EL4‐IL2 adhered per endothelial cell. Means of three biological replicates, *P < .05. J, K, Mechanism of activated PD1+ NK cells (EL4IL2) to endothelial cells (MBMMEC). Normoxic DiO‐stained EL4IL2 adhered to MBMMEC in a 5 to 1 ratio for 20 min at 20°C (J) or 4°C (K). Blocking by anti‐PD‐L1Fc silent, chimeric, and blocking mouse IgG1 was performed by incubating the MBMMEC endothelial cell culture for 1 h with 10 µg/mL anti‐PD‐L1 IgG1 before EL4IL2 cell addition, either at room temperature (J) or 4°C (K). The results are expressed as R = the number of EL4IL2 per one MBMMEC. Data are the mean values from five mice from three separate experiments *P < .05

Figure 7 shows the expression of PD‐1 on the surface of the EL4 cell line and its IL2 activated subline EL4‐IL2, which were chosen as models for NK and activated NK cells, respectively (Figure 7A,B). The expression of PD‐L1 and PD‐L2 was studied on the surface of organospecific murine microvascular endothelial cells from either the brain (MBrMEC) or the bone marrow (MBMMEC) microvasculature, chosen to be representative of metastatic and proangiogenic endothelial cell recruitment sites, respectively. PD‐L2 was not detected, whereas PD‐L1 expression was induced on MBMMECs (Figure 7C) but it was poorly modulated by hypoxia on MBrMECs (Figure 7C). The direct effect of hypoxia on the induction of PD‐L1 expression was controlled by the concomitant induction of the *vegf‐a* (Figure 7F) and *vhl* (Figure 7E) genes at the RNA level in both MBrMEC and MBMMEC lines, and confirmed at the protein level by ELISA measurement (Figure 7F).

The molecular mechanism regulating the adhesion between activated PD1+NK cells and hypoxic PD‐L1+ endothelial cells (Figure 7J,K) was tested for the involvement of immune checkpoint molecules in the adhesion process as quantified by flow cytometry.^45^ Blocking with an anti‐PD‐L1 neutralizing antibody significantly reduced the adhesion of EL4IL2 to MBMMEC, by 35% (Figure 7K). This partial inhibition was obtained when the adhesion experiment was performed at 4°C, whereas at room temperature (20°C) no effect was observed. The bivalent IgG1 antibody, although devoid of active Fc, may activate the energy‐dependent adhesion process (Figure 7J), inducing an adhesion molecule cascade and masking the role of individual partners.

### 3.7 Modulation of the tumour microenvironment: chemokine receptor expression in response to vessel normalization by ITPP

The intercellular interactions between immune and non‐immune stromal cells at the tumour site occur through a fundamental cross‐talk ensured by chemokines and their receptors. The metastatic process has largely been demonstrated for CXCL12 and its receptor CXCR4, and CCL21 and its receptor CCR7.

**FIGURE 8:**
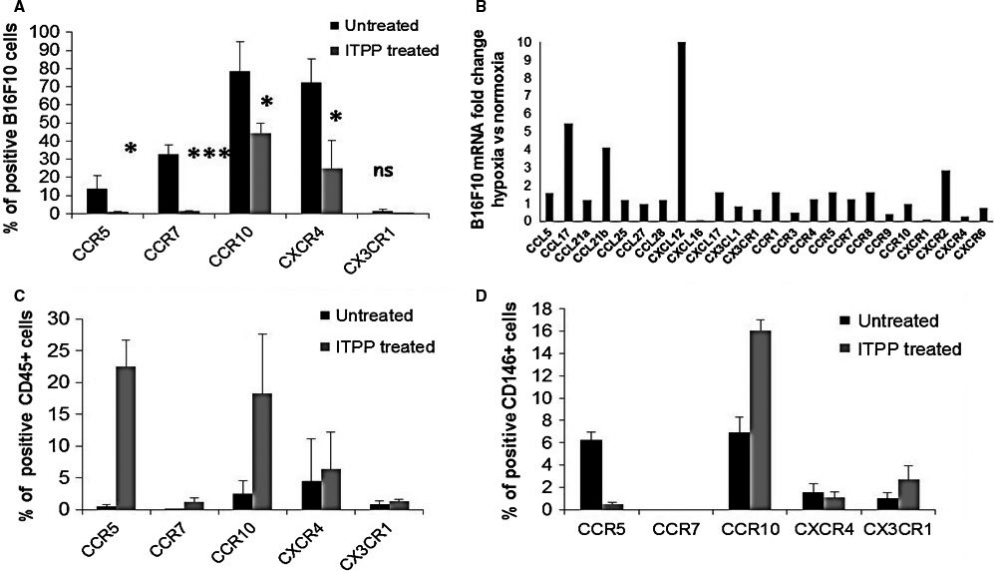
Chemokine receptor modulation of expression on tumour cells and immune and endothelial enriched cell populations by ITPP treatment–induced vessel normalization. A, Flow cytometry detection of the expression of chemokine receptors on B16F10 cells in the tumour site. B, RT‐qPCR quantification of the mRNA relative expression of chemokines and receptors by B16F10 cells in hypoxia as compared to normoxia. Data represent the mean values from five experimental mice of one representative experiment out of five. C, Flow cytometry detection of the expression of chemokine receptors on CD45+ immune cells in the tumour site. D, Flow cytometry detection of the expression of chemokine receptors on the endothelial enriched cell population in the tumour site. Data represent the means of five experimental mice from three separate experiments (*P < .05, **P < .01, ***P < .0005)

Figure 8 demonstrates the strong effect that ITPP treatment exerted on chemokine receptor expression in the tumour cell population, but also in immune cells and endothelial cells at the tumour site.

A radical effect of ITPP treatment on CCR5 expression was observed on tumour cells (Figure 8A), corroborating the inverse effect observed on the CD45+ population (Figure 8C) and the M1 macrophage repolarization effect.^16^

The CCR7/CCL21 axis is responsible for melanoma cell metastases into lymph nodes^11^, ^16^, ^49^ and promotes tumorigenesis by stem cells.^50^ A total reduction in CCR7 expression was observed on tumour cells (Figure 7A), whereas no significant expression of this receptor was detected in either immune or endothelial cells (Figure 8C,D).^51^

The proportion of B16F10 cells expressing CCR10, the receptor for CCL27 (cutaneous T‐cell attracting chemokine), was diminished upon ITPP treatment, whereas the proportion of immune cells (*R* = 10) (Figure 8C) and the endothelial cell‐enriched population (*R* = 2) (Figure 8D) increased. This axis may control the recruitment of immunocompetent cells.^52^

Tumour cells expressing the receptor for CXCL12 (CXCR4), which is involved in metastasis,^53^ were strongly reduced (Figure 8A), reflecting the effect of hypoxia on tumour cells,^54^ whereas CXCR4 expression was not affected in CD45+ cells (Figure 8C) or in the endothelial cell‐enriched population (Figure 8D).

Moreover, the endothelial enriched cell‐population selectively displayed an induced expression of the fractalkine receptor, CX3CR1, upon ITPP treatment (Figure 8D), which corresponds with the reoxygenation effect and vessel restoration.^54^

Comparison of the expression of chemokine and chemokine receptors at the mRNA level in tumour cells in hypoxia and normoxia (Figure 8B) indicated a strong effect of hypoxia on the chemokine components of the microenvironment. This is highly significant for chemokines, as CCL17, which promotes cancer, is dependent on hypoxia.^55^ The major changes appear in the induction of mRNAs for chemokines CXCL12 and CCL21b, confirming the role of hypoxia in the metastatic process of melanoma cells.

## 4 DISCUSSION

The present knowledge of tumours and modern tumour‐treatment strategies is leading researchers to design new and combined approaches. From promising data on the role of angiogenesis, the possibility of creating new means for treating tumours has arisen and is being actively explored.^38^

Early anti‐angiogenesis treatments were often devoted to blocking VEGFs and associated receptors by the use of sophisticated monoclonal antibodies.^56^ Clinical studies point to the delicate balance that should be reached to enhance the efficacy of treatments.^57^

These approaches have shown limitations due to the resistance processes that tumours develop, in terms of cell selection under pressure from the tumour microenvironment.

Studies on niches favouring the resistance of cancer cells have clarified the means of cell selection leading to cancer stem‐like cells. Also called cancer‐initiating cells, they can reproduce tumour diversity.^58^

As pathologic angiogenesis originates from the hypoxic cell milieu and acidification following the process of anaerobic glycolysis, anoxia is reached when efficient anti‐angiogenic drugs, such as monoclonal antibodies,^59^ are used and participate in creating conditions of strong resistance in quiescent cells, which can survive and give tumour populations highly aggressive properties.^59^ The main goal is thus to obtain treatments capable of compensating for hypoxia in the tumour.^3^ Based on the rationale that a non‐hypoxic tumour will no longer sustain pathologic angiogenesis and allow vessels to be normalized and functional, new treatment approaches are attempting to reach stabilization of normalized angiogenesis.^60^

As previously described, pO_2_ elevation creates important modifications in the tumour microenvironment, namely in parameters responsible for tumour immunosuppression: the main reason for treatment pitfalls.

Our two main approaches, through which we demonstrated that it is possible to normalize vessels in tumours, have also shown the ability to stably maintain normalization. Our approaches are complementary, and based on the restoration of oxygen tension. Counteracting the excess VEGF produced in hypoxia, which acts as an efficient chemoattractant for immunosuppressive cells,^43^, ^61^ our hypoxia‐conditioned cell‐targeted gene therapy allows the fine‐tuning of therapeutic angiogenesis‐based treatment.^45^ The treatment efficiently reduced tumour size in both melanoma and mammary carcinoma.^43^, ^45^ The second strategy, based on direct modulation of O2 release, uses myo‐inositol trispyrophosphate, which directly allows for higher delivery of oxygen by red blood cells.^28^ The ability of the molecule to activate PTEN was shown to ensure stable vessel normalization and function.^30^ This leads to reductions in the number of tumours and cancer stem cells. Both approaches are non‐toxic for both tumour and normal cells. Consequently, analysing their effects in the context of tumour‐stromal changes and the host reaction was the purpose of this work. We showed here that in conditions which permit a 50% reduction in tumour size and in which the vasculature is normalized, the tumour microenvironment is deeply transformed. The main transformation concerns the immune cell composition inside the tumour. NK cells are the most reactive cells and were more numerous and better able to penetrate the tumour mass after treatment. This work indicates the role of PD1/PD‐L1 interactions.

This might be attributed to the strong effect of ITPP on CD31+ cells. Indeed, our data indicate that the proportions of endothelial cells increased upon ITPP treatment, and they express the junction molecule CD31, which is increased in normoxia compared to hypoxia^46^ and is a criterion of functional vessels.^30^

Following previous evidence that endothelial PD‐L1 is hypoxia‐dependent,^19^, ^62^ we showed that, upon expression on endothelial cells during hypoxia, it can block the passage of PD1+ NK cells and prevent penetration into the tumour mass. Indeed, inhibition of the adhesion of NK cells‐to‐bone marrow‐derived endothelial cells was observed upon neutralization by PD‐L1 activity. This mechanism is energy‐dependent and corroborates the effect of ITPP in vivo. Indeed, ITPP treatment–induced normalization reduced PD‐L1 and PD‐L2 expression on tumour cells, as well as on CD31+ and CD45+ cells. Interestingly, it increased PD1+ expression on CD45+ cells, similar to NK cells. This correlates with the reduction of other cells that are PD1+ such as Tregs, which could suppress the tumour immune response. Consequently, ITPP treatment reduces the PD1/PD‐L1/PD‐L2 immune checkpoint activity. As PD1 expressing cells are not reduced, but PD‐L1 and PD‐L2 are less expressed, the resulting effect is a reduction in the programmed death‐mediated elimination of the immunocompetent and actively tumour killing cells (mainly cytotoxic T cells and NK cells). Importantly, the reduction in MDSCs cooperates with the reduction in Th2 cells to restore the immune response, creating an inflammatory state.

As we demonstrated previously, vessel normalization strongly controls metastases^30^; this work, thus, focussed on the modulation of chemokines and their receptors. We confirmed the control of the main chemokine/receptor axes, CXCL12/CXCR4^63^ and CCL21/CCR7,^11^, ^64^ involved in tumour escape and verified their dependency on hypoxia.

This work demonstrates that hypoxia compensation in the tumour can be reached by novel means for vessel normalization. It primarily provides evidence that stable vessel normalization induces drastic changes in the tumour stroma, and these changes result in the restoration of the tumour immune response through the influence of hypoxia‐dependent molecules, such as the immune checkpoint molecules PD1/PD‐L1 and PD‐L2. Activated NK cells (CD49b+ CD226+)^46^ were increased, and NK cells were able to invade the tumour, unlike in non‐treated tumours, in which they remained in the vessels. Indeed, non‐treated endothelial cells express PD‐L1 during hypoxia, which was reduced by an increase in pO_2_, and was obtained here upon ITPP treatment. Moreover, the Treg cell proportion is considerably reduced; thus, it cannot account for the increase in CD45+PD1+ cells^47^ reported here among the whole tumour population, both in melanoma and mammary carcinoma.

As confirmed by the finding that there are other immune checkpoints, such as CTLA4 and CD47, the expression of which is HIF‐1 regulated,^27^ and the role of the vessel normalization effect of ITPP treatment and the reduction in tumour stem cells also previously seen upon ITPP treatment,^30^ this method provides a powerful new means for controlling the immunological balance in the tumour, whereas avoiding the side effects associated with systemically blocking molecules, such as PD1 on NK cells or CD8+ T cells^65^ and PD‐L1 on T regulators.^27^, ^65^ Stable vessel normalization therapies create novel options for designing combined antitumor strategies based on the immunological adjuvant effect, which can help avoid the pitfalls faced by other cancer therapies.

## CONFLICT OF INTEREST

CK is a shareholder in NormOxys Inc.

## AUTHOR CONTRIBUTIONS

**Bouchra El Hafny‐Rahbi:** Conceptualization (equal); data curation (equal); formal analysis (supporting); investigation (equal); methodology (equal); project administration (supporting); supervision (equal); validation (supporting); visualization (equal). **Klaudia Brodaczewska:** Data curation (supporting); formal analysis (supporting); investigation (supporting); supervision (supporting). **Guillaume Collet:** Conceptualization (equal); data curation (equal); methodology (equal); validation (equal). **Aleksandra Majewska:** Data curation (equal); formal analysis (equal); investigation (supporting); methodology (equal). **Krzysztof Klimkiewicz:** Data curation (supporting); formal analysis (supporting); investigation (supporting); methodology (supporting); validation (equal); visualization (equal). **Anthony Delalande:** Data curation (equal); formal analysis (equal); investigation (supporting); methodology (equal); validation (supporting); visualization (equal); writing – original draft (supporting). **Catherine Grillon:** Data curation (supporting); formal analysis (supporting); methodology (supporting). **Claudine Kieda:** Conceptualization (lead); data curation (lead); formal analysis (lead); funding acquisition (lead); investigation (lead); methodology (lead); project administration (lead); supervision (lead); validation (lead); visualization (lead); writing – original draft (lead); writing – review and editing (lead).

## Supporting information

Figure S1‐S4

## Data Availability

The data that support the findings of this study are available from the corresponding author upon reasonable request.
