# Supplementary material for: Tumour angiogenesis normalized by myo‐inositol trispyrophosphate alleviates hypoxia in the microenvironment and promotes antitumor immune response
Source: J Cell Mol Med. 2021 Feb 23;25(7):3284–99. doi: 10.1111/jcmm.16399 (PMC8034441; doi:10.1111/jcmm.16399)
Supplement: Supplementary file 1 — Figure S1‐S4 [file JCMM-25-3284-s001.docx]

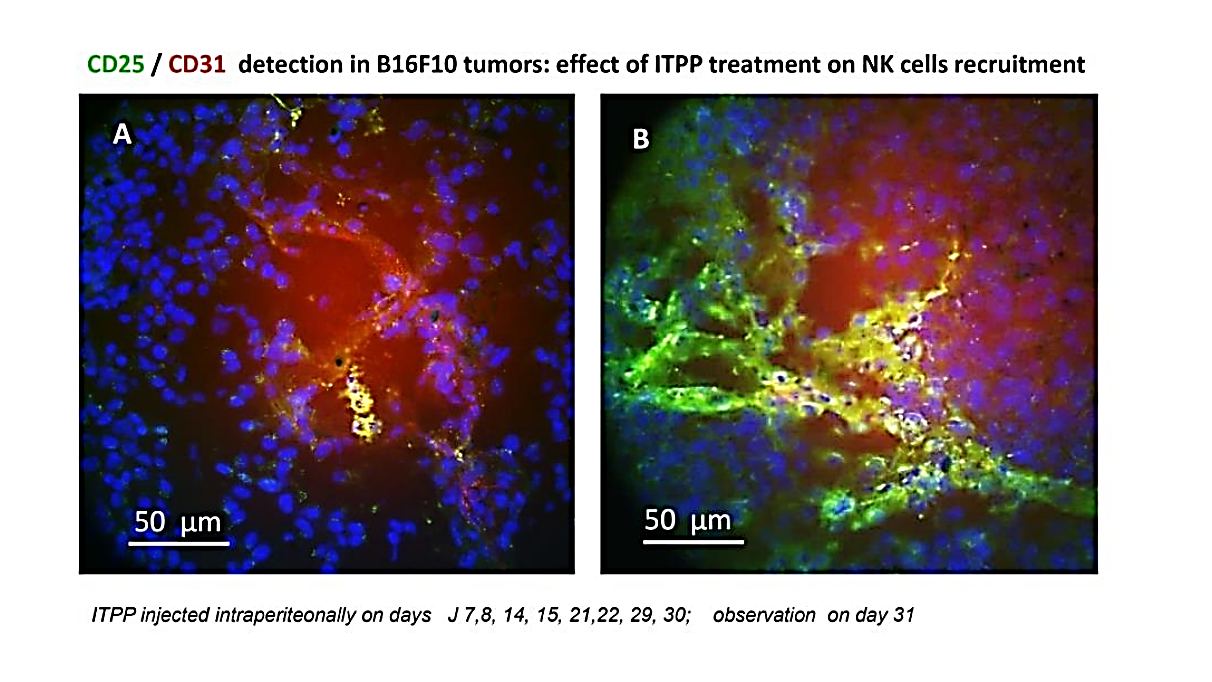

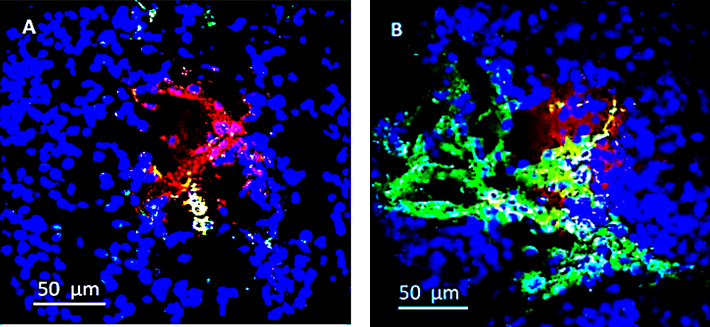


Supplementary Figure S1


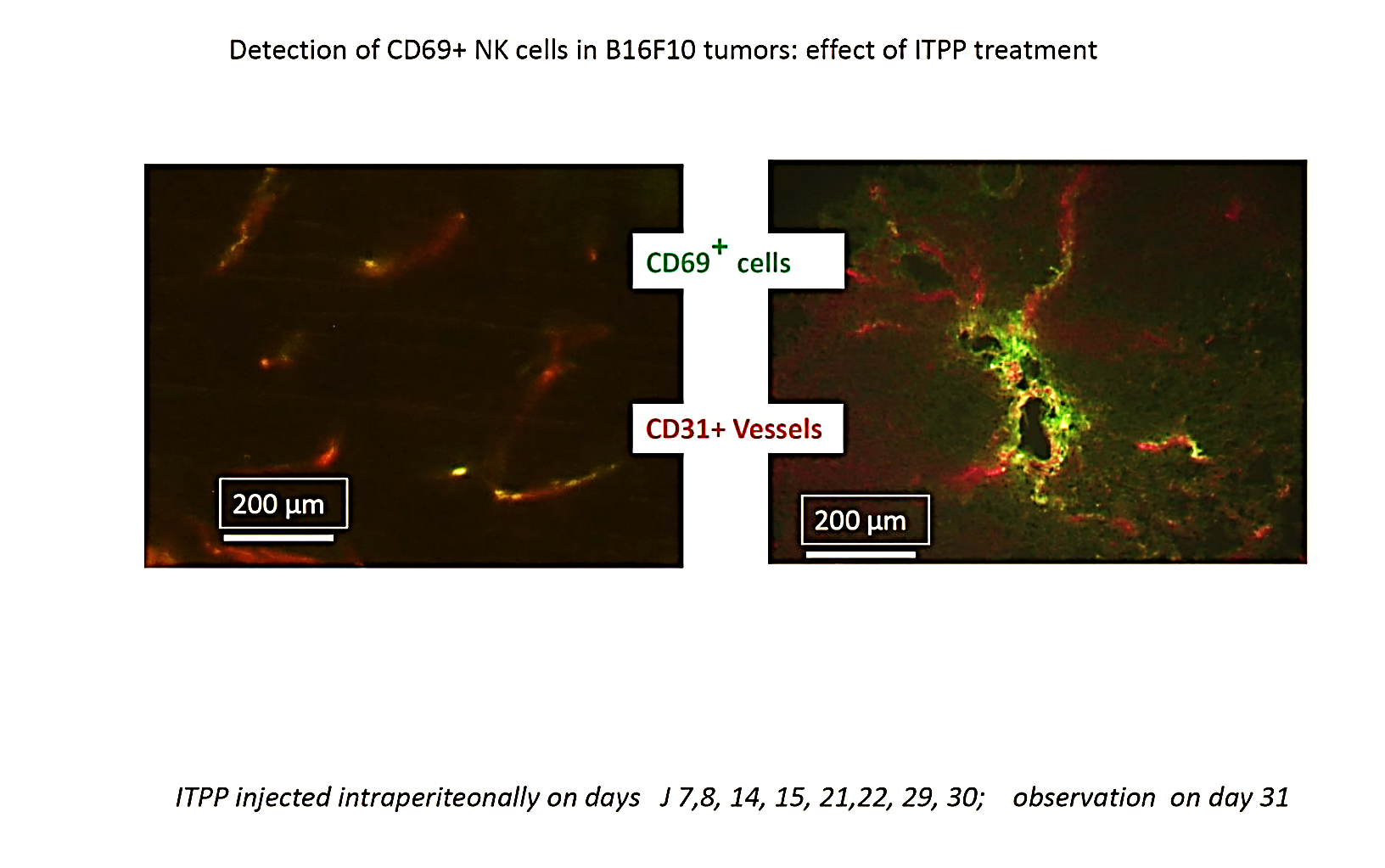


Supplementary Figure S2


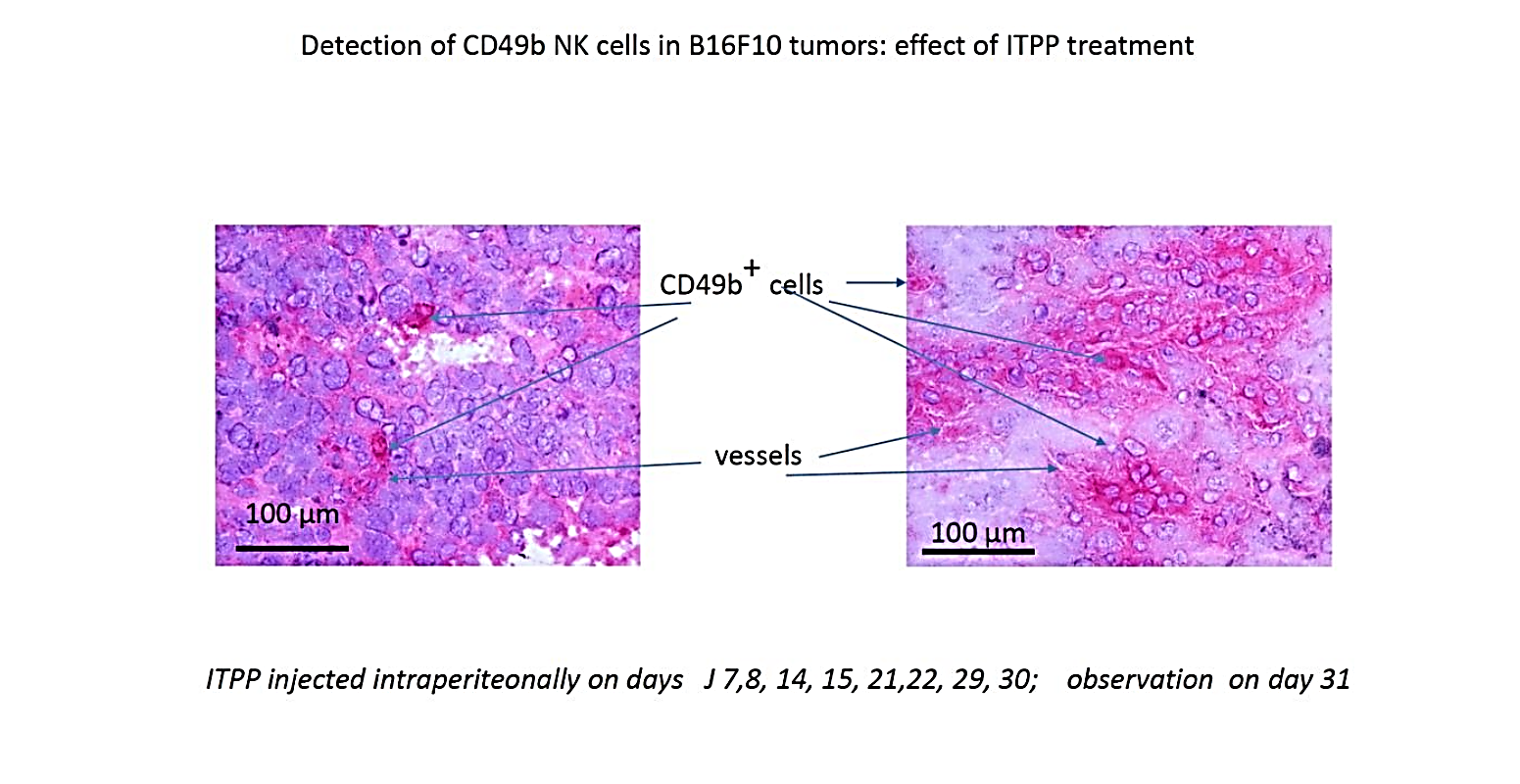


Supplementary Figure S3


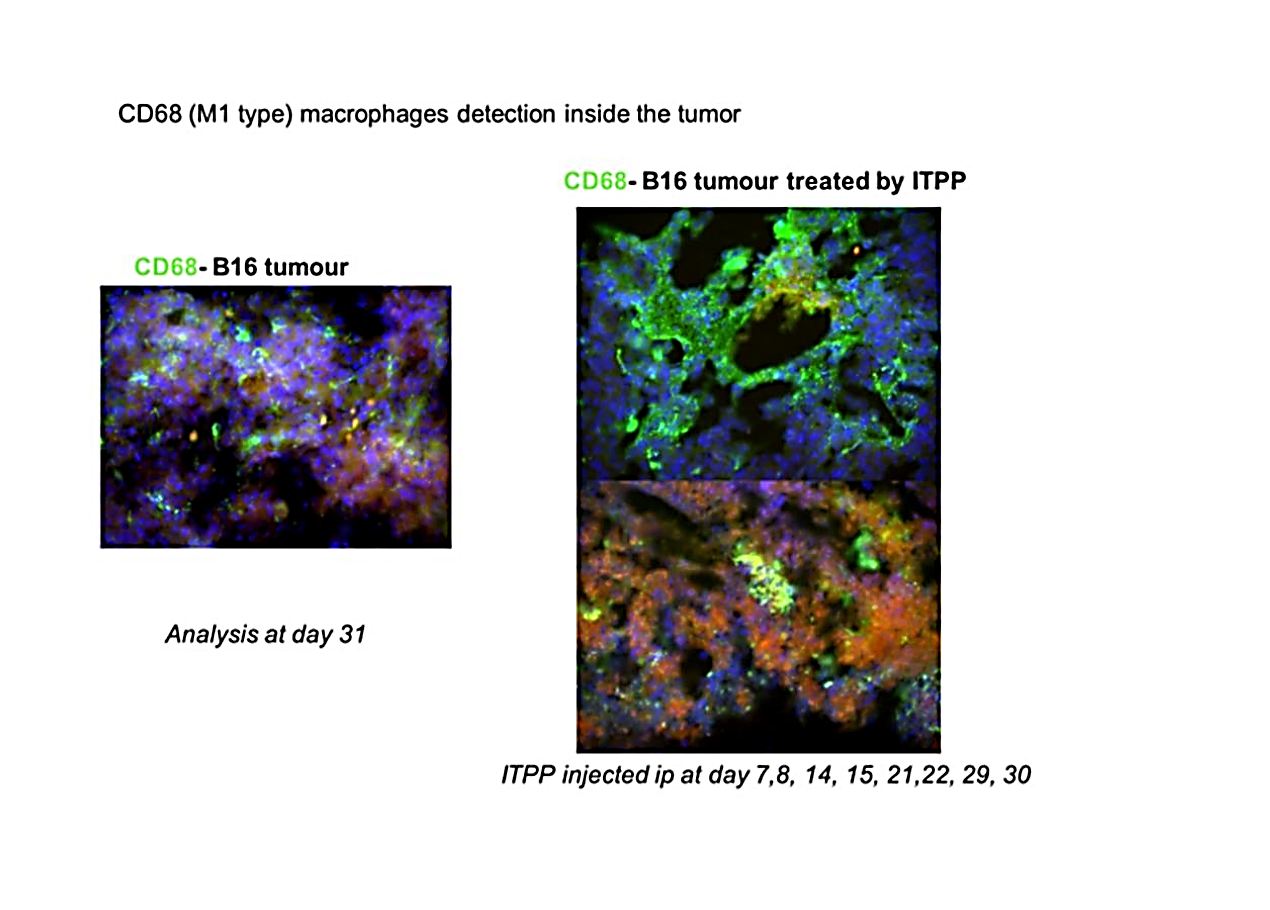


Supplementary Figure S4
